# Antitumor Compounds from Marine Actinomycetes

**DOI:** 10.3390/md7020210

**Published:** 2009-06-11

**Authors:** Carlos Olano, Carmen Méndez, José A. Salas

**Affiliations:** Departamento de Biología Funcional e Instituto Universitario de Oncología del Principado de Asturias (I.U.O.P.A), Universidad de Oviedo, 33006 Oviedo, Spain; E-Mails: olanocarlos@uniovi.es (C.O.); cmendezf@uniovi.es (C.M.)

**Keywords:** anthracycline, indolocarbazole, macrolide, non-ribosomal peptide synthetase, polyketide synthase

## Abstract

Chemotherapy is one of the main treatments used to combat cancer. A great number of antitumor compounds are natural products or their derivatives, mainly produced by microorganisms. In particular, actinomycetes are the producers of a large number of natural products with different biological activities, including antitumor properties. These antitumor compounds belong to several structural classes such as anthracyclines, enediynes, indolocarbazoles, isoprenoides, macrolides, non-ribosomal peptides and others, and they exert antitumor activity by inducing apoptosis through DNA cleavage mediated by topoisomerase I or II inhibition, mitochondria permeabilization, inhibition of key enzymes involved in signal transduction like proteases, or cellular metabolism and in some cases by inhibiting tumor-induced angiogenesis. Marine organisms have attracted special attention in the last years for their ability to produce interesting pharmacological lead compounds.

## 1. Introduction

Actinomycetes, characterized by a complex life cycle, are filamentous Gram-positive bacteria belonging to the phylum *Actinobateria* that represents one of the largest taxonomic units among the 18 major lineages currently recognized within the domain Bacteria [1]. *Actinobacteria* are widely distributed in terrestrial and aquatic ecosystems, especially in soil, where they play a crucial role in the recycling of refractory biomaterials by decomposing complex mixtures of polymers in dead plant, animal and fungal materials. They are important in soil biodegradation and humus formation by the recycling of nutrients associated with recalcitrant polymers such as keratin, lignocelluloses and chitin [2–4] and produce several volatile substances like geosmin responsible of the characteristic “wet earth odor” [5]. They also exhibit diverse physiological and metabolic properties, such as the production of extracellular enzymes [3,6].

Around 23,000 bioactive secondary metabolites produced by microorganisms have been reported and over 10,000 of these compounds are produced by actinomycetes, representing 45% of all bioactive microbial metabolites discovered [7]. Among actinomycetes, around 7,600 compounds are produced by *Streptomyces* species [7]. Many of these secondary metabolites are potent antibiotics, which has made streptomycetes the primary antibiotic-producing organisms exploited by the pharmaceutical industry [7]. Members of this group are producers, in addition, of clinically useful antitumor drugs such as anthracyclines (aclarubicin, daunomycin and doxorubicin), peptides (bleomycin and actinomycin D), aureolic acids (mithramycin), enediynes (neocarzinostatin), antimetabolites (pentostatin), carzinophilin, mitomycins and others [8,9]. However, the search for novel drugs is still a priority goal for cancer therapy, due to the rapid development of resistance to multiple chemotherapeutic drugs. In addition, the high toxicity usually associated with cancer chemotherapy drugs and their undesirable side effects increase the demand for novel antitumor drugs active against untreatable tumors, with fewer side effects and/or with greater therapeutic efficiency [10].

Progress has been made recently on drug discovery from actinomycetes by using high-throughput screening and fermentation, mining genomes for cryptic pathways, and combinatorial biosynthesis to generate new secondary metabolites related to existing pharmacophores [11]. In addition, in the last years the isolation of marine actinomycetes has been a great source of new compounds and their isolation all around the globe, from shallow costal sediments to the deepest sediments from the Mariana Trench, demonstrates that actinomycetes are ubiquitous in marine sediments, but at lower numbers than in soil [12–24]. The oceans are highly complex environments and house a diverse assemblage of microbes that occur in environments with extreme variations in pressure, salinity, and temperature. The oceans cover around 70% of the Earth’s surface and present themselves as an unexplored area of opportunity. Marine microorganisms encompass a complex and diverse assemblage of microscopic life forms, of which it is estimated that only 1% has been cultured or identified [25]. In addition, marine actinomycetes have been found in symbiosis with different marine invertebrates, especially sponges [26,27]. Marine actinomycetes have attracted great attention since they have developed unique metabolic and physiological capabilities that not only ensure survival in extreme habitats, but also offer the potential to produce compounds with antitumor and other interesting pharmacological activities that would not be observed in terrestrial microorganisms [28–32], perhaps because of their close relationships with marine eukaryotic organisms including mammals [11,26]. However, one of the main problems associated with marine actinomycetes is the difficulty often found for their culture, due to specific requirements like sea salt, since in some cases these microorganisms are obligate halophiles [33]. There are a number of reports on techniques and approaches for accessing previously uncultured soil actinomycetes and the biosynthesis gene clusters they harbor [34,35]. In the case of marine actinomycetes these studies are only beginning, but there have already been several attempts to optimize their isolation and growth from several sources [26,36,37] as well as the improvement of the fermentation process for the production of specific compounds [33,38,39] and the development of tools to facilitate the genetic manipulation of the isolated biosynthesis gene clusters [40,41].

In this review we focus on novel antitumor compounds identified from marine actinomycetes and classified them in terms of their chemical structure (Table 1), covering the literature until February 2009.

## 2. Polyketides

Polyketides are a large family of natural products produced by step-wise decarboxylative Claisen-type condensation of acyl-CoA precursors, reactions catalyzed by polyketide synthases (PKSs). The carbon skeleton of polyketides may be further reduced and modified based on the programming encoded by different domains present in PKSs with ketoreductase, dehydratase and enoylreductase activities. Three types of PKSs are known to date: type I PKSs are multifunctional enzymes organized into modules, type II PKSs are multienzyme complexes carrying out a single set of activities and type III PKSs, also known as chalcone synthase-like PKSs, are iteratively acting condensing enzymes [42,43].

**Figure 1:**
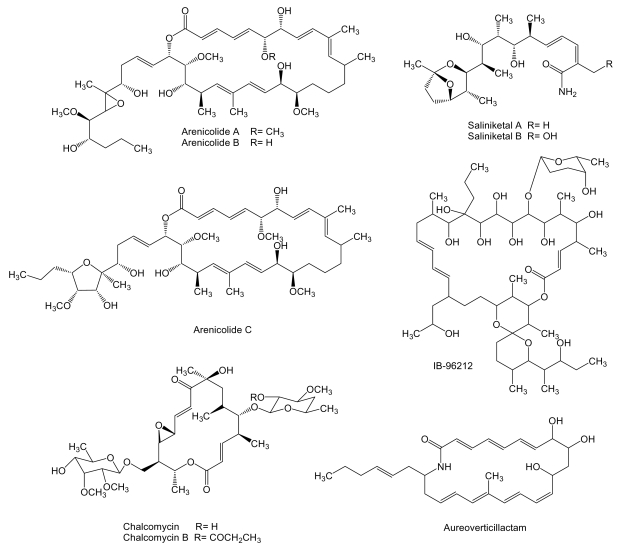
Polyketide compounds arenicolides and saliniketals produced by *S. arenicola* CNR-005 and other macrolides and macrolactams.

A high number of type I polyketide derived compounds with antitumor activity have been isolated from marine actinomycetes. Such is the case of arenicolides (Figure 1), 26-membered polyunsaturated macrolactones, produced by the obligate marine actinomycete *Salinispora arenicola* strain CNR-005 isolated from a marine sediment sample collected at a depth of 20 m from the coastal water around the island of Guam.

In particular, arenicolide A was found to exhibit moderate cytotoxicity toward the human colon adenocarcinoma cell line HCT-116 with an IC_50_ of 30 μg/mL [44]. Two bicyclic polyketides, saliniketal A and B (Figure 1), were also isolated from the same strain of *S. arenicola*. Using cultures of human bladder carcinoma T24 cells in conjunction with terephthalic acid, a potent tumor promoter that induces ornithine decarboxylase (ODC), saliniketals were found to inhibit ODC induction with IC_50_ values of 1.95 and 7.83 μg/mL, respectively [45]. ODC, an important target for the chemoprevention of cancer, is a directed transcriptional target of the oncogene *myc* and is overexpressed in various tumor cells [46].

Several macrolactones have been reported in addition to the arenicolides (Figure 1). IB-96212, a 26-membered macrolide that contains a spiroketal lactone structure, is produced by *Micromonospora* sp. L-25-ES25-008 isolated from a sponge collected at the Indian Ocean near the coast of Mozambique [47]. This compound showed cytotoxic activity against mouse leukemia P-388 and human lung non-small cell A-549, colon adenocarcinoma HT-29 and melanoma MEL-28 cell lines. The activity against P-388 cell line was four orders of magnitude higher than the activity against A-549, HT-29 and MEL-28 cell lines [48]. Chalcomycin, a 16-membered macrolide, is produced by *Streptomyces* sp. M491 isolated from the Qingdao coast (China) [49]. In addition, chalcomycin and the related compound chalcomycin B have been isolated from *Streptomyces* strain B7064 found in mangrove sediments in Hawaii [50]. Chalcomycin has been found to inhibit protein synthesis in HeLa human cervix carcinoma cell line [51]. The biosynthesis gene cluster of this compound was isolated and characterized from *S. bikiniensis* [52]. Aureoverticillactam is a 22-membered macrocyclic lactam produced by *S. aureo-verticillatus* NPS001583 isolated from marine sediments. Aureoverticillactam was found to possess moderate growth inhibitory activity against human colorectal adenocarcnioma HT-29, Jurkat leukemia and mouse melanoma B16F10 cell lines [53].

**Figure 2:**
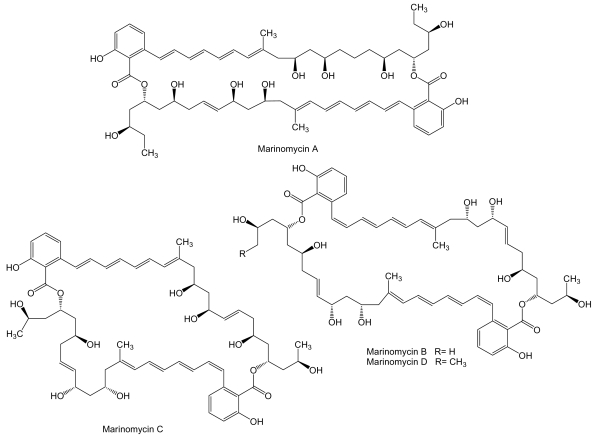
Structures of marinomycins.

To a novel class of polyketides belong marinomycins (Figure 2), unusual macrodiolides composed of dimeric 2-hydroxy-6-alkenyl-benzoic acid lactones with conjugated tetraene-pentahydroxy polyketide chains, produced by *Marinispora* sp. CNQ-140 isolated from a sediment sample collected at a depth of 56 m offshore of La Jolla, California. These compounds inhibit cancer cell proliferation with an average LC_50_ of 0.2–2.7 μM against the NCI’s 60 cancer cell line panel. Marinomycin A showed significant tissue type selectivity being more active against human melanoma cell lines LOX IMVI, M14, SK-MEL-2, SK-MEL-5, UACC-257, and UACC-62. The most sensitive strain was melanoma SK-MEL-5 with an LC_50_ of 5.0 nM. Marinomycins B and C also showed potent activities with average LC_50_ values of 0.9 and 0.2 μM, respectively. The potent and selective antitumor activity of these compounds suggests a specific, but as yet unknown, mechanism of action [54].

**Figure 3:**
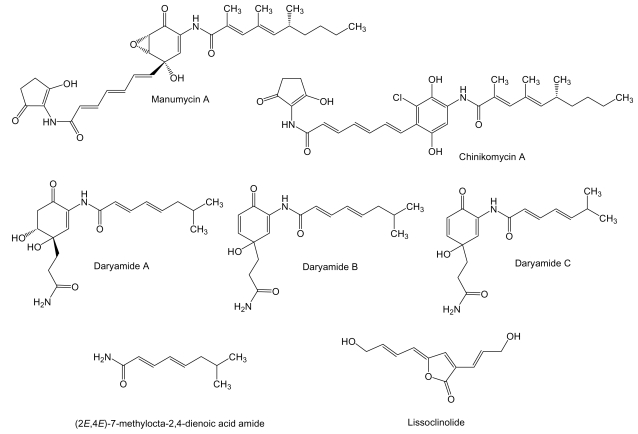
Structures of manumycin type compounds and lissoclinolide.

The manumycins (Figure 3) constitute a class of compounds with antibiotic, cytotoxic, and other biological activities. It has been reported that manumycin A and its analogues inhibit Ras farnesyl transferase and the growth of *Ki*-ras-activated murine fibrosarcoma in mice [55,56]. The side chains in manumycins appear to be a typical polyketide-derived moiety, differing with respect to their combinations of starter and elongation units. The central cyclohexene ring may be derived from the polyketide as in the case of manumycins or from some modified amino acid like 3-amino-5-hydroxybenzoic acid. Manumycin A and chinikomycins A and B (the quinone form of chinikomycin A) were isolated from *Streptomyces* sp. M045 derived from sediment of Jiaozhou Bay in China. Chinikomycins A and B showed moderate antitumor activity. Chinicomycin A selectively inhibited proliferation in cell lines of mammary cancer MAXF 401NL (IC_50_ of 2.41 μg/mL), melanoma MEXF 462NL (IC_50_ of 4.15 μg/mL), and renal cancer RXF 944L (IC_50_ of 4.02 μg/mL). Chinikomycin B showed selective antitumor activity against the mammary cancer cell line MAXF 401NL (IC50 of 3.04 μg/mL) [57]. Daryamides belong also to the manumycin family of compounds. They were isolated from *Streptomyces* strain CNQ-085 obtained from marine sediment collected at a depth of 50 m off San Diego coast, California. Daryamides A to C and (2*E*,4*E*)-7-methylocta-2,4-dienoic acid amide were subjected to cytotoxicity evaluation against the human colon carcinoma cell line HCT-116 showing that daryamide A exhibited significantly more potent cancer cell cytotoxicity, with an IC_50_ of 3.15 μg/mL, than daryamides B and C [58].

Even though it is not produced by a marine actinomycete we include in this review the antitumor compound lissoclinolide (Figure 3) isolated from the marine ascidian *Lissoclinum patella* [59]. This compound was originally isolated from a fungus and a soil actinomycete [60,61], which points to an actinomycete associated to *L. patella* as the true source of lissoclinolide. This compound, a small non-nitrogenous lactone with a putative polyketide origin probably synthesized by a type I PKS, was able to inhibit cell growth in various mammalian tumor lines at an average IC_50_ of 395 nM, and in particular the human colon tumor COLO 205, HCC-2998, HCT-116 and HCT-15 cell lines which resulted in a strong arrest in the G_2_/M phase of the cell cycle [62].

**Figure 4:**
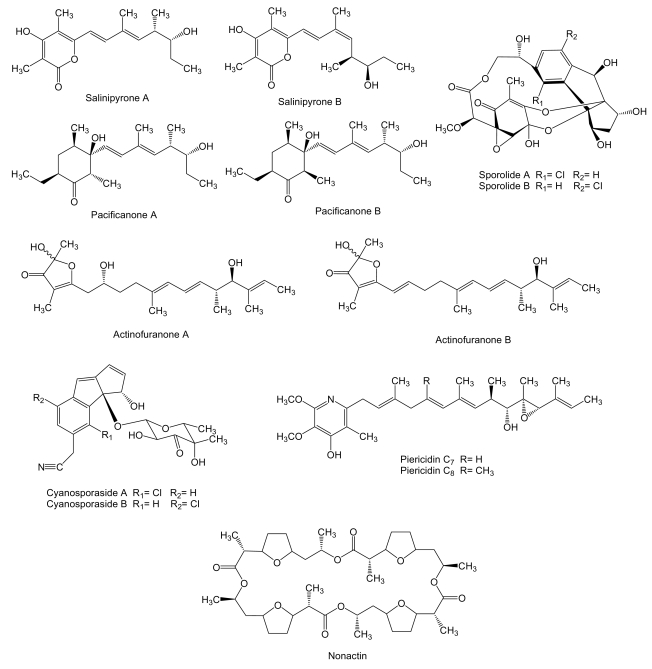
Structures of salinipyrones, pacificanones, sporolides, actinofuranones, cyanosporasides and piericidins type I polyketides and nonactin type II polyketide.

Four new polyketides (Figure 4), salinipyrones (A and B), and pacificanones (A and B) have been isolated from cultures of the obligate marine actinomycete *Salinispora pacifica* CNS-237 found in a sediment sample collected in the Palau island, western Pacific Ocean. The biological activity of these compounds is currently being examined in diverse bioassays. In initial screening, the salinipyrones and the pacificanones displayed no significant activity in a cancer cytotoxicity assay using HCT-116 human colon cancer cells. In an isolated mouse splenocyte model of allergic inflammation, salinipyrone A displayed moderate inhibition of interleukin-5 production by 50% at 10 *μ*g/mL without measurable human cell cytotoxicity [63]. The same apparent lack of biological activity has been found also for the polyketides sporolide A and B (Figure 4) produced by *Salinispora tropica* strain CNB-392 isolated from marine sediments near Chub Cay (Bahamas) [64] and actinofuranones A and B (Figure 4) isolated from culture extract of *Streptomyces* strain CNQ766 derived from a sediment sample collected from Guam [65]. Sporolides displayed no activity against human colon carcinoma HCT-116 cells and showed no significant antibiotic activity [64]. Actinofuranones showed weak *in vitro* cytotoxicity against mouse splenocyte T-cells and macrophages with IC_50_ values of 20 μg/mL and were inactive against human colon carcinoma HCT-116 cells [65]. Cyanosporasides A and B (Figure 4) are cyclopenta[*a*]indene glycosides structurally related to sporolides and proposed to be cyclization products of an enediyne precursor probably synthesized by an iterative type I PKS [42]. They have been isolated from the culture broth of *S. pacifica* strain CNS103 isolated from sediments collected at a depth of 500 m in Palau island. To date, limited testing has shown that cyanosporaside A has weak cytotoxicity against human colon carcinoma HCT-116 (IC_50_ 30 μg/mL) [66].

Piericidins C_7_ and C_8_ (Figure 4), probably synthesized by type I PKS, are produced by *Streptomyces* sp. YM14–060 isolated from an unidentified ascidian collected at Iwayama Bay, Palau Island [67]. The biological activity of piericidins was examined using rat glial cells transformed with the adenovirus E1A gene (RG-E1A-7), Neuro-2a mouse neuroblastoma cells, C6 rat glioma cells and 3Y1 rat normal fibroblast. The adenovirus E1A gene product inactivates the retinoblastoma tumor suppressor protein that plays an important role in cell-cycle and apoptosis control in mammalian cells and is inactivated during the development of a wide variety of cancers [68]. Piericidins C_7_ and C_8_ showed selective cytotoxicity against RG-E1A-7 cells (IC_50_ of 1.5 nM and 0.45 nM, respectively) and inhibited the growth of Neuro-2a cells (IC_50_ of 0.83 nM and 0.21 nM, respectively) without cytotoxic cell death. On the other hand, C6 rat glioma cells and 3Y1 rat normal fibroblast were not affected by piericidins [69].

Nonactin (Figure 4), a cyclic polyether also known as macrotetrolide, has been isolated from cultures of *Streptomyces* sp. KORDI-3238 isolated from a deep-sea sediment sample collected at Ayu Trough in the western Pacific Ocean [70]. The biosynthesis gene cluster of nonactin has previously been isolated and characterized from *S. griseus* DSM40695 [71] revealing that it is synthesized by a non-iteratively acting type II PKS that involves five ketosynthases and lacks the acyl carrier protein. Nonactin has been shown to be an effective inhibitor against the multidrug-resistant human erythroleukemia cell line K-562 [72].

**Figure 5:**
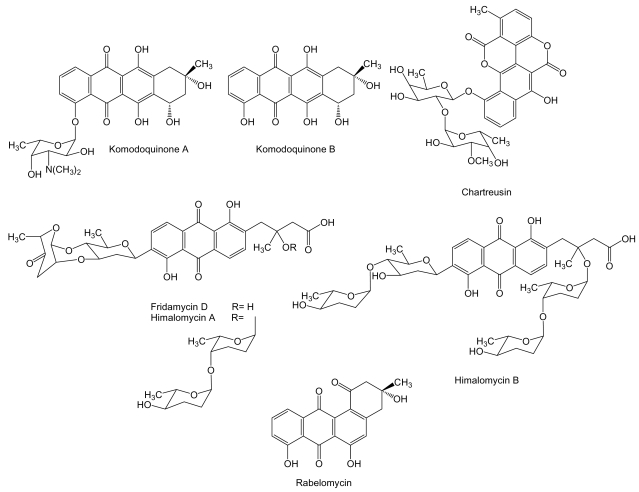
Structures of several anthracyclines and anthracycline-like compounds.

Aromatic polyketides are synthesized by type II PKS and are further divided into different structural classes such as anthracyclines, angucyclines and tetracyclines among others. To the anthracycline family of compounds belongs komodoquinone A, and its aglycone, komodoquinone B (Figure 5) produced by *Streptomyces* sp. KS3 isolated from marine sediments. Komodoquinone A is a unique anthracycline, in which a previously unknown amino sugar is attached to C4 instead to C7 as in the most known anthracyclines [73]. Komodoquinone A was found to induce neuronal cell differentiation in the neuroblastoma cell line Neuro-2a at a concentration of 1 μg/mL, an activity not shown by doxorubicin. Treatment with komodoquinone A arrested Neuro-2a cells at the G_1_ phase while these treated with adriamycin were arrested at the G_2_/M phase. These data suggested that the amino sugar moiety attached to C4 might be important for neuritogenic activity of komodoquinone A, activity probably exerted by a mechanism different from the intercalation in DNA [73,74]. Another anthracycline related compound, chartreusin, has been isolated from cultures of *Streptomyces* sp. QD518 isolated from the Jiaozhou Bay of Quindao, China [75]. Chartreusin (Figure 5) is an aromatic glycosylated polyketide, currently in phase II clinical trials [76], that possesses an unusual bislactone synthesized through anthracycline intermediates that might undergo a series of oxidative rearrangements to generate the final bislactone structure. This particular biosynthetic process has been unraveled by the isolation of the chartreusin biosynthesis gene cluster from *S. chartreusi*s [77]. Chartreusin has been shown to exert antitumor activity through binding to DNA, radical-mediated single-strand breaks and inhibition of topoisomerase II [78]. It possesses a significant chemotherapeutic activity against various tumor cell lines such as murine P388 and L1210 leukemia, and B16 melanoma cells [79]. *Streptomyces* sp. B6921 is the producer of several anthracycline C-glycosides such as fridamycin D, himalomycin A and B, and the angucycline rabelomycin (Figure 5). Only antibiotic activity has been reported for these compounds [80]. However, other compounds analogous to fridamycin D and himalomycin A and B such as vineomycins have been shown to exhibit antitumor activity against Sarcoma-180 solid tumor in mice [81].

**Figure 6:**
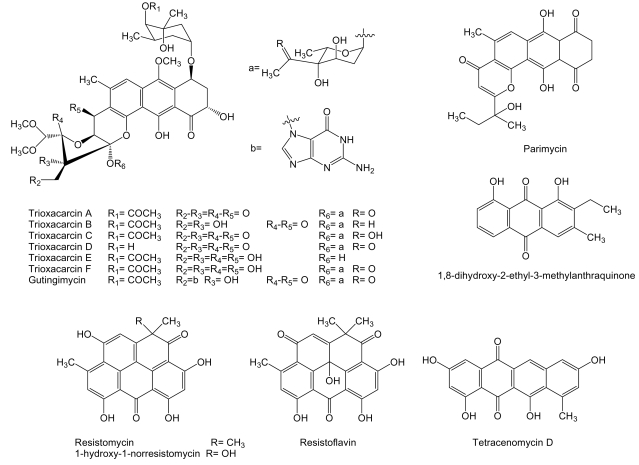
Structures of anthraquinone and quinone-related compounds.

Several compounds of the anthraquinone family (Figure 6), closely related to anthracyclines, are produced by *Streptomyces* sp. isolate B8652 derived from a sediment of the Laguna de Términos at the Gulf of México. All these compounds, parimycin, trioxacarcins and gutingimycin, showed antitumor activities at different degrees [82–84]. It has been shown that trioxacarcin A forms a stable complex with dsDNA and the cleavage of these complexes provided the natural product gutingimycin by guanine abstraction [85]. Parimycin showed activity against human tumor cell lines of stomach cancer GXF 251L, lung cancer H460, LXFA 629L, and LXFL 529L, breast cancer MCF-7 and MAXF 401NL, melanomas MEXF 462NL and MEXF 514L with IC_70_ values ranging from 0.9 to 6.7 μg/mL [82]. Trioxacarcins A to D showed pronounced antitumor activities with mean IC_70_ values ranging from 0.001 to 2.161 μg/mL against human tumor cell lines of colon cancer HT-29, melanoma MEXF 514L, lung adenocarcinoma LXFA 526L, large cell lung cancer LXFL 529L and H-460, central nervous system SF-268, mammary cancer MCF-7, prostate cancer PC3M, and renal cancer RXF 631L. Trioxacarcin A was the most potent of all trioxacarcins, and gutingimycin was the less active with a mean IC_70_ value of 3.386 μg/mL. Trioxacarcin A also proved to be very toxic in preliminary *in vivo* experiments in tumor bearing nude mice, being the maximum tolerated dose between 0.1 and 0.3 mg/kg. Trioxacarcins B to D showed selective antitumor activity against certain tumor cell lines [83]. Another compound from the same family, 1,8-dihydroxy-2-ethyl-3-methylanthraquinone, has been identified from cultures of *Streptomyces* sp. FX-58, which was isolated from marine plant *Salicornia herbacea* collected in Qingdao, Shandong province, China. This compound showed inhibitory effect against human tumor cell lines of pro-myelocytic leukemia HL-60, gastric carcinoma BGC-823 and adenocarcinoma MDA-MB-435 with IC_50_ of 6.83, 82.2 and 56.59 μg/mL, respectively [86].

Tetracenomycin D and other quinone-related compounds with antitumor activity have been isolated from different marine acinomycetes (Figure 6). *S. chinaensis* AUBN_1_/7 isolated from marine sediment samples of Bay of Bengal, India, is the producer of 1-hydroxy-1-norresistomycin and resistoflavin [87]. These compounds together with resistomycin and tetracenomycin D are produced by *Streptomyces* sp. B8005 isolated from sediments of the Laguna de Términos at the Gulf of México [88]. Resistomycin was isolated, in addition, from *Streptomyces* sp. B4842 from mud sediment of a coastal site of Mauritius, Indian Ocean [88]. Tetracenomycin D was previously showed to possess cytotoxic activity [89] and it has been isolated from *S. glauscescens* as an intermediate in the biosynthesis of tetracenomycin D, whose biosynthesis gene cluster has been studied and used for the generation of several derived compounds [90]. Resistomycin, among other activities, has also been proposed to be a modulator of apoptosis [91]. The biosynthesis gene cluster of resistomycin has been isolated and characterized from *S. resistomycificus*, and, as expected, involves a type II PKS [92]. 1-hydroxy-1-norresistomycin [87] and resistoflavin [93] showed cytotoxic activity against human gastric adenocarcinoma HMO2 and hepatic carcinoma HePG2 cell lines.

**Figure 7:**
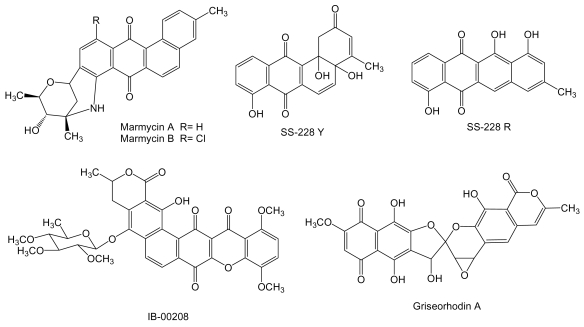
Structures of angucyclines and related compounds.

Two new cytotoxic quinones of the angucycline class, marmycins A and B (Figure 7) were isolated from the culture broth of *Streptomyces* strain CNH990 isolated from a sediment sample collected at a depth of 20 m at the entrance to the Sea of Cortez, 5 km east of Cabo San Lucas, México [94]. In cytotoxic assays using the human cell line of colon adenocarcinoma HCT-116, marmycin A showed an IC_50_ of 60.5 nM, almost 18 times more potent than marmycin B, which showed an IC_50_ of 1.09 μM. Marmycin A was further evaluated for its *in vitro* cytotoxicity showing a mean IC_50_ value of 0.022 μM against 12 human tumor cell lines (breast, prostate, colon, lung, leukemia). In the same assays marmycin B was significantly less potent with a mean IC_50_ value of 3.5 μM [94]. SS-228 Y (Figure 7) is an angucycline type compound [95] produced from a species of *Chainia* (now *Streptomyces*) isolated from shallow sea mud in Sagami Bay. During SS-228 Y structural characterization this compound was found to be very labile to light and heat, being converted into the anthracycline like compound SS-228 R [95,96]. SS-228 Y inhibits growth of Ehrlich breast adenocarcinoma in mice. A prolongation of the survival period was observed in mice inoculated with Ehrlich ascites tumor when SS-228 Y was administered for 10 days at doses of more than 1.56 μg per day. The acute toxicity of SS-228 Y, shown by LD_50_, in mice was between 1.56 and 6.25 mg/kg by intraperitoneal injection [97].

IB-00208 (Figure 7) is a polycyclic xanthone related to angucyclines and other aromatic polyketides [98]. It was isolated from *Actinomadura* sp BL-42-PO13-046 collected in the northern coast of Spain associated to a polychaete. IB-00208 showed a potent cytotoxic activity against mouse leukemia P-388 and human lung non-small cell A-549, colon adenocarcinoma HT-29 and melanoma SK-MEL-28 cell lines [99]. Griseorhodin A (Figure 7) [100], that belongs to the family of pentangular polyphenols related to angucyclines and other aromatic polyketides, is an inhibitor of human telomerase [101], a ribonucleoprotein complex involved in the stabilization of telomere length in stem cells and reproductive cells and found in almost all human tumors but not in adjacent normal cells [102]. The biosynthesis gene cluster for griseorhodin A has been isolated and characterized from *Streptomyces* sp. JP95 associated with the marine ascidian *Aplidium lenticulum* collected at Heron Island, Queensland, Australia [103].

## 3. Non-Ribosomal Peptides

This class of natural products comprises peptides synthesized by non-ribosomal peptide synthetases (NRPS). The amino acid monomers incorporated by NRPS assembly lines are aminoacyl-AMP mixed anhydrides that follow the same chemical logic as PKSs for chain elongation and are then modified based on the program encoded by different domains present in NRPS modules, which can include epimerization, methyltransferase, reductase or oxidase activities. Quite often non-ribosomal peptides also contain some unique structural features such as heterocyclic elements and deoxysugars [104–106].

**Figure 8:**
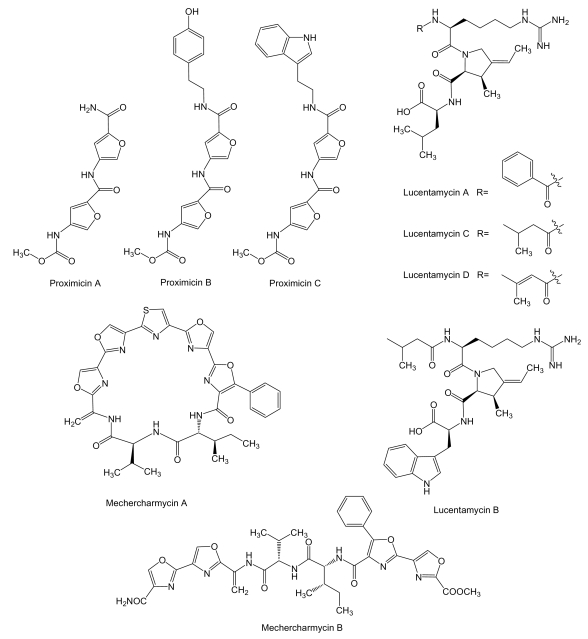
Structures of proximicins, lucentamycins and mechercharmycins.

Proximicins (Figure 8) are aminofuran antibiotics, probably synthesized by a NRPS system, produced by *Verrucosispora* strain MG-37 and *V. maris* AB-18–032, and isolated from sediments collected at a depth of 250 m in the Raune Fjord, Norway and the Sea of Japan at a depth of 289 m, respectively [107,108]. Proximicins A, B and C showed significant growth inhibitory activities towards human gastric adenocarcinoma AGS (GI_50_ of 0.6, 1.5 and 0.25 μM, respectively) and hepatocellular carcinoma Hep G2 (GI_50_ of 0.82, 9.5 and 0.78 μM, respectively) [107,109], and were found to induce arrest AGS cells in G_0_/G_1_ and to increase the levels of p53 and p21 [109]. Lucentamycins (Figure 8), 3-methyl-4-ethylideneproline-containing peptides, are produced by *Nocardiopsis lucentensis* strain CNR-712 isolated from sediment collected from a shallow saline pond on the island of Little San Salvador, in the Bahamas. Lucentamycins A and B showed significant *in vitro* cytotoxicity against human colon carcinoma HCT-116 cell line with IC_50_ values of 0.20 and 11 μM, respectively. However, lucentamycins C and D were not cytotoxic in the same assay suggesting that the presence of an aromatic ring is essential for the biological activity of this class of compounds [110]. Mechercharmycins (Figure 8) are produced by *Thermoactinomyces* sp. YM3-251, isolated from sea mud collected at Mecherchar in the Republic of Palau (North Pacific Ocean). While mechercharmycin A showed cytotoxic activity against human lung adenocarcinoma A549 and Jurkat leukemia cells with IC_50_ values of 0.04 μM, mechercharmycin B did not show inhibitory activity in these assays even at 1 μM, which suggests the cyclic structure of mechercharmycin A must be essential for the antitumor activity [111].

**Figure 9:**
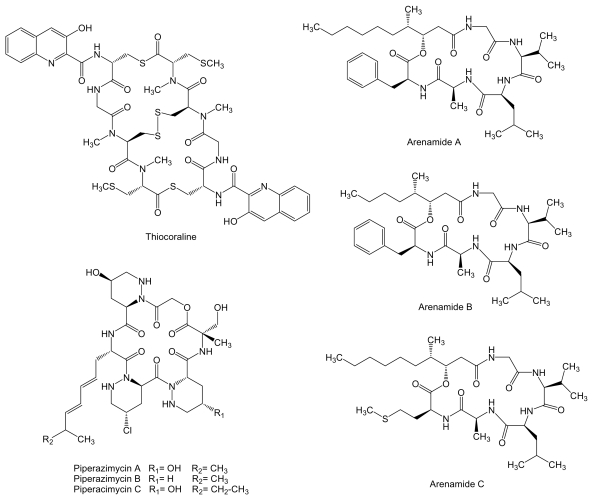
Structures of depsipeptides thiocoraline, arenamides and piperazimycins.

Thiocoraline (Figure 9) is a 2-fold symmetric bicyclic non-ribosomally synthesized octathiodepsipeptide, characterized by the presence of peptide and ester bonds, produced by *Micromonospora* sp. L-13-ACM2-092 isolated from a soft coral collected at the Indian Ocean near the coast of Mozambique [112,113]. It shows potent antitumor activity against murine leukemia P388, human lung adenocarcinoma A549, and melanoma MEL288, being the activity against these cell lines 5-fold more potent than for human colon adenocarcinoma HT-29 cell line (IC_50_ values of 0.002 vs. 0.01 μM, respectively) [112]. In addition, on both LOVO and SW620 human colon cancer cell lines, thiocoraline caused an arrest in G_1_ phase of the cell cycle and a decrease in the rate of S phase progression towards G_2_/M phases, due to its DNA bisintercalative properties and its DNA polymerase α inhibition [114]. Thiocoraline is structuraly related to other bisintercalator compounds of the quinoxaline family with potential therapeutic applications like echinomycin, inhibitor of hypoxia-inducible factor-1 that controls genes important for tumor progression and metastasis that are involved in glycolysis, angiogenesis, migration, and invasion [115]. The biosynthesis gene cluster of thiocoraline has been isolated, characterized and heterologously expressed in *S. lividans* and *S. albus* [116].

Three new cyclohexadepsipeptides, arenamides (Figure 9), were isolated from the fermentation broth of a marine bacterial strain identified as *S. arenicola* CNT-088 which was obtained from a marine sediment sample collected at a depth of 20 m off the Great Astrolab Reef, in the Kandavu Island chain, Fiji. Arenamides A and B exhibited weak in vitro cytotoxicity against human colon carcinoma HCT-116 with IC_50_ values of 13.2 and 19.2 μg/mL, respectively [117].

In addition, arenamides have been associated to chemoprevention of carcinogenesis by suppression of NF*κ*B activation. NF*κ*B regulates the expression of a number of genes, the products of which are involved in tumorigenesis [118,119]. The effect of arenamides on NF*κ*B activity was studied with stably transfected 293/NF*κ*B-Luc human embryonic kidney cells induced by treatment with tumor necrosis factor (TNF). Arenamides A and B blocked TNF-induced activation in a dose- and time-dependent manner with IC_50_ values of 3.7 and 1.7 *μ*M, respectively [117]. Piperazimycins (Figure 9) are cyclic hexadepsipeptides isolated from the fermentation broth of a *Streptomyces* sp. strain CNQ-593, cultivated from marine sediments samples collected at a depth of approximately 20 m near the island of Guam. Antitumor activities for piperazimycins were initially evaluated *in vitro* against the human colon carcinoma HCT-116 cell line. All compounds exhibited significant cytotoxicity with an average GI_50_ of 76 ng/mL for each. Piperazimycin A also showed potent biological activity when evaluated against the NCI’s cancer cell line panel, with mean GI_50_, TGI and LC_50_ values for all cell lines of 100 nM, 300 nM and 2 μM, respectively. Overall, piperazimycin A exhibited a nearly 3-fold more potent activity against solid tumors (average LC_50_ of 13.9 μM) than against the leukemia cell lines tested (average LC_50_ of 31.4 μM), being most active against the melanoma (average LC_50_ of 0.3 μM), central nervous system (average LC_50_ of 0.4 μM), and prostate cell lines (average LC_50_ of 0.6 μM) cancers [120].

## 4. Mixed Polyketide-Nonribosomal Peptides

As mentioned above, NRPSs and type I PKSs are multifunctional proteins that are organized into modules and use a similar strategy for the assembly of these two distinct classes of natural products. The combined use of NRPSs and type I PKSs allows assembling hybrid polyketide/non-ribosomal peptide compounds derived from amino acids and short-chain carboxylic acids.

**Figure 10:**
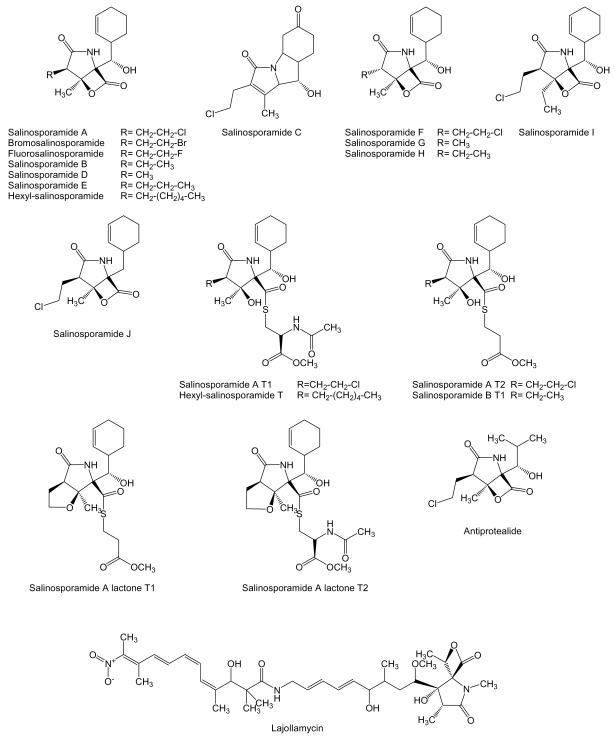
Structures of mixed polyketide/non-ribosomal peptide compounds.

The most known compound of this family is salinosporamide A (Figure 10), a highly potent inhibitor of the 20S proteasome currently in phase I clinical trials for the treatment of cancer [76]. It is produced by *S. tropica* strain CNB-392 isolated from a sediment sample collected at a depth of about 1 m from a mangrove environment in Chub Cay, Bahamas [121] and other *S. tropica* strains like CNB-440 and CNB-476 isolated also from Bahamas [122,123]. Salinosporamide A displayed potent *in vitro* cytotoxicity against human colon carcinoma HCT-116 with an IC_50_ value of 11 ng/mL, and was shown potent and highly selective in the NCI’s 60 cell line panel. The greatest potency was observed against human cell lines of non-small cell lung cancer NCI-H226, central nervous system cancer SF-539, melanoma SK-MEL-28, and breast cancer MDA-MB-435 (all with LC_50_ values less than 10 nM). When salinosporamide A was tested against purified rabbit muscle 20S proteasome, a multicatalytic complex responsible for degrading most intracellular proteins in eukaryotes, it inhibited proteasomal chymotrypsin-like proteolytic activity with an IC_50_ value of 1.3 nM [121]. The 26S proteasome, composed of 19S and 20S components, is a multicatalytic complex responsible for degrading most intracellular proteins in eukaryotes. Three distinguishable proteolytic activities are localized in the 20S proteasome and are classified as chymotrypsin-, caspase-, and trypsin-like, all of them inhibited by salinosporamide A, in particular chymotrypsin and caspase-like [124]. Compounds of the same family, salinosporamides B-J (Figure 10), have been isolated together with salinosporamide A from *S. tropica* [125–127]. In addition, new derivatives of salinosporamide A were produced by replacement of synthetic sea salt with sodium bromide in the fermentation media of *S. tropica* that led to the production of bromosalinosporamide [128], and by derivatization of salinosporamide A and B leading to the production of several thioester analogues (designed as T in Figure 10) [127]. Salinosporamide A thioester derivatives T1 and T2 were found potent 20S proteasome inhibitors with IC_50_ values of 3.6-and 1.5-fold higher than salinosporamide A [127]. The characterization of the salinosporamide A biosynthesis gene cluster during the sequencing of *S. tropic*a CNB-440 circular genome [122] has allowed the generation of several derivatives such as fluorosalinosporamide produced by mutasynthesis using a chlorinase *salL* mutant [129], and antiprotealide (Figure 10) produced by a combination of genetic engineering and precursor-directed biosynthesis [130]. Antiprotealide has been recently found as a natural product produced by different strains of *S. tropica* [131].

Lajollamycin (Figure 10) is another mixed polyketide/non-ribosomal peptide produced by *S. nodosus* strain NPS007994 isolated from marine sediments collected in Scripps Canyon, La Jolla, California. Lajollamycin was found to inhibit the growth of murine melanoma cell line B16-F10, with an EC_50_ of 9.6 μM [132]. The biosynthesis gene cluster of oxazolomycin, a structural close relative of lajollamyin, has been isolated and characterized from *S. albus* JA3453 confirming the hybrid peptide-polyketide origin of these compounds [133].

## 5. Isoprenoids

Isoprenoids, similar to terpenes, are one of the largest families of natural compounds. Isoprenoids are derived from five-carbon isoprene units assembled and modified in different ways. They are classified into several groups based on the number of C5 units that form part of their structure: monoterpenes (C10), sesquiterpenes (C15) and diterpenes (C20) [134].

**Figure 11:**
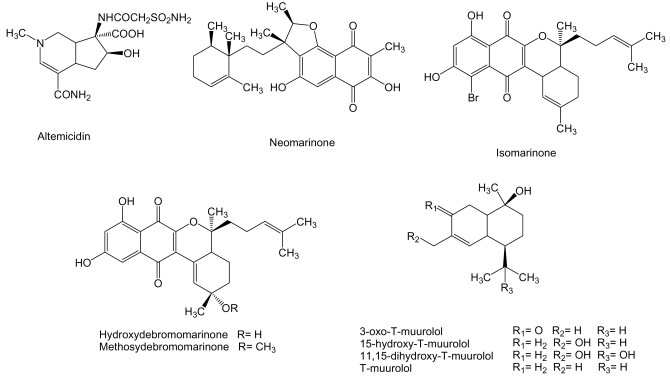
Structures of monoterpenes and sesquiterpenes isolated from marine actinomycetes.

Altemicidin (Figure 11) with a monoterpene-alkaloid skeleton is produced by *S. sioyaensis* SA-1758 isolated from sea mud collected at Gamo, Miyagi Prefecture, Japan [135]. This compound was found to inhibit the growth of murine lymphoid leukemia L1210 and carcinoma IMC cell lines with IC_50_ values of 0.84 and 0.82 μg/mL, respectively, although it showed high acute toxicity in mice [136].

Marinones (Figure 11) are sesquiterpenoid naphthoquinones with a mixed polyketide-terpenoid origin [137]. Neomarinone, isomarinone, hydroxydebromomarinone and methoxydebromomarinone were produced by actinomycete isolate CNH-099 obtained from a sediment sample taken at 1 m depth in Batiquitos Lagoon, North of San Diego, California. These compounds showed moderate *in vitro* cytotoxicity, IC_50_ of 8 μg/mL, against human colon carcinoma HCT-116 cells. In addition, neomarinone generated a mean IC_50_ value of 10 μM in the NCI’s 60 cancer cell line panel [138,139]. T-muurolol sesquiterpernes (Figure 11) were isolated from *Streptomyces* strain M491 derived from sediment obtained from Jiaozhou Bay, Qingdao coast, China [49,140]. Seven out of eight sesquiterpenes of the T-muurolol family were tested for their cytotoxycity against 37 human tumor cell lines but, except for 15-hydroxy-T-muurolol which was moderately cytotoxic with an IC_50_ of 6.7 μg/mL, the other compounds including 3-oxo-T-muurolol, 11,15-dihydroxy-T-muurolol, T-muurolol and 3α-hydroxy-T-muurolol showed no activity [140].

**Figure 12:**
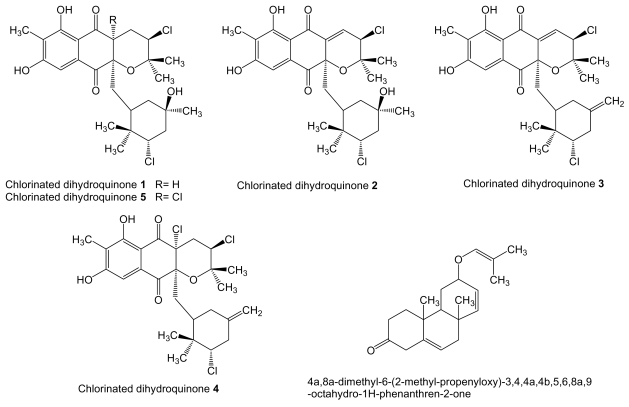
Structures of chlorinated dihydroquinones and active compound from strain MS1/7.

Several chlorinated dihydroquinones (**1–5**, Figure 12), with a mixed terpenoid/polyketide origin, are produced in saline culture of the actinomycete strain CNQ-525, isolated from ocean sediments collected at a depth of 152 m near La Jolla, California [141]. Dihydroquinones **4** and **5** were identical to the previously reported antibiotics A80915A and A80915C produced by soil actinomycete *S. aculeolatus* [142]. Dihydroquinones **1**, **2** and **4** were found to be cytotoxic toward human colon carcinoma HCT-116 cells with IC_50_ of 2.4, 0.97 and 1.84 μg/mL, respectively [141].

*Actinobacterium* sp. MS1/7 isolated form sediments taken at the Bay of Bengal is the producer of compound 4a,8a-dimethyl-6-(2-methyl-propenyloxy)-3,4,4a,4b,5,6,8a,9-octahydro-1H-phenanthren-2-one (Figure 12), with putative isoprenoid origin. This compound was found to inhibit the growth by 54% of human leukemia HL-60 cell line at 0.05 μg/mL and to possess a reduced toxicity against non-tumor cells since only 2.3% of murine erythrocytes and 1.6% of human erythrocytes were lysed at concentrations of 35–40 μg/mL [143].

## 6. Indolocarbazoles

**Figure 13:**
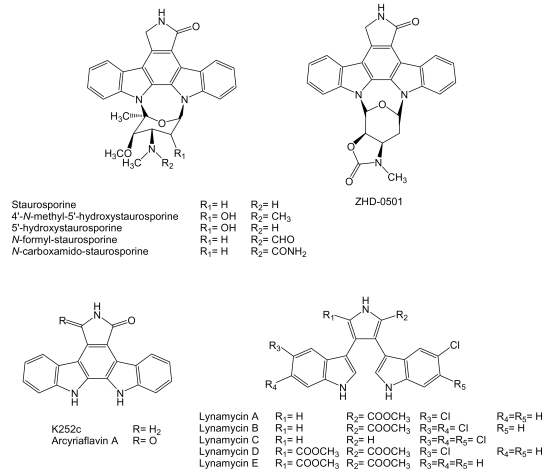
Structures of indocarbazoles and bisindole pyrroles produced by marine actinomycetes.

Most of the compounds of this family contain a characteristic indolo[2,3-*a*]pyrrolo[3,4-*c*]carbazole core derived from two units of tryptophan, with sugars attached derived from glucose and methionine. These compounds constitute a separate type of antitumor drugs with several mechanisms of action including DNA-damage targeting on topoisomerases I and II, and inhibition of protein kinases, including serine/threonine and tyrosine kinases [144]. One of the better known indolocarbazole, staurosporine (Figure 13) has been isolated from several marine actinomycetes including komodoquinones-producer *Streptomyces* sp. KS3 [74] after its discovery from cultures of *S. staurosporeus* AM-2282 [145]. The staurosporine biosynthesis gene cluster was identified and characterized from *Streptomyces* sp. TP-A0274 and *S. longisporoflavus* [146,147], which has led to the generation of several derivatives of indolocarbazoles staurosporine and rebeccamycin with antitumor activity [147–150]. Staurosporine has been isolated, in addition, together with two natural analogues, 4′-*N*-methyl-5′-hydroxystaurosporine and 5′-hydroxystaurosporine (Figure 13) from *Micromonospora* sp. L-31-CLCO-002 obtained from a homogenate of the sponge *Clathrina coriacea* collected on the Coast of Fuerteventura Island in the Canary Islands archipelago [151].

The cytotoxic activities of these indolocarbazoles were determined *in vitro* in cell cultures of murine macrophague P388D_1_ (IC_50_ of 0.01, 0.02 and 0.04 μg/mL), human lung adenocarcinoma A549 (IC_50_ of 0.0005, 0.002 and 0.004 μg/mL), colon adenocarcinoma HT-29 (IC_50_ of 0.02, 0.004 and 0.004 μg/mL) and melanoma SK-MEL-28 cell lines (IC_50_ of 0.001, 0.002 and 0.004 μg/mL). Staurosporine showed the strongest activity against P388D_1_, A549 and SK-MEL-28 cell lines, while 4′-*N*-methyl-5′-hydroxystaurosporine and 5′-hydroxystaurosporine were more active against HT-29 cell line [151]. *N*-formyl- and *N*-carboxamido-staurosporines have been isolated from cultures of *Streptomyces* sp. QD518 isolated from the Jiaozhou Bay of Quindao, China [75]. *N*-formyl- and *N*-carboxamido-staurosporines were tested *in vitro* for antitumoral activity in a panel of 37 human tumor cell lines derived from solid human tumors. *N*-carboxamido-staurosporine was found to be the most potent compound with a mean IC_50_ and IC_70_ values of 0.016 and 0.17 μg/mL, respectively, while *N*-formyl-staurosporine showed also high activity with mean IC_50_ and IC_70_ values of 0.063 and 0.37 μg/mL, respectively [75]. ZHD-0501 (Figure 13) is a novel naturally occurring staurosporine analog carrying a heterocycle fused to the pyran ring produced by *Actinomadura* sp. 007, strain isolated from a sea sediment sample collected in Jiaozhou Bay, China [152]. ZHD-0501 was shown to inhibit the proliferation of human lung adenocarcinoma A549, hepatocarcinoma BEL-7402, and pro-myelocytic leukemia HL60 cancer cell lines and mouse leukemia P388 cells with the inhibition rates of 82.6%, 57.3%, 76.1%, and 62.2% at 1 μM, respectively. It also inhibited the proliferation of mouse mammary cancer tsFT210 cells, by inhibiting the cell cycle at the G_2_/M phase, with the inhibition rates of 28.3% at 21 μM and 20.5% at 2.1 μM [152].

Two known indolocarbazole alkaloids with anticancer properties, K252c (staurosporine aglycon) and arcyriaflavin A (Figure 13), are produced by marine actinomycete strain Z_2_039-2, isolated from the sea sediment collected on the coast of Qingdao, China. K252c was found to induce 57.3% apoptosis on culture of human chronic myelogenous leukemia K562 cell line at 10 μM and nearly 100% at 100 μM. In the same assay arcyriaflavin A induced 25.9% apoptosis at 10 μM and up to 68.93%, at 100 μM [153]. K252c and arcyriaflavin A have been found, in addition, to reduced the relative resistance of cells transfected with ABCG2, a transporter with potential importance in cancer drug resistance, to SN-38 (7-ethyl-10-hydroxycamptothecin) in cytotoxicity assays [154]. Several analogues of K252c and arcyriaflavin A have been generated using genes involved in rebeccamycin biosynthesis [149].

Lynamicins (Figure 13) bisindole pyrroles are related to bisindolylmaleimides and indolocarbazoles due to their close structural and biosynthetic relationships [144], are produced by *Marinispora* sp. NPS12745 isolated from a marine sediment collected off the coast of San Diego, California. These compounds have been only tested for antimicrobial activity showing good potency against staphylococci and enterococci [155]. However, there are reports of bisindolylmaleimide derivatives with potent antitumor and antiangiogenic properties [144] and with potential to reduce resistance mediated by transporter ABCG2 [154].

## 7. Others

**Figure 14:**
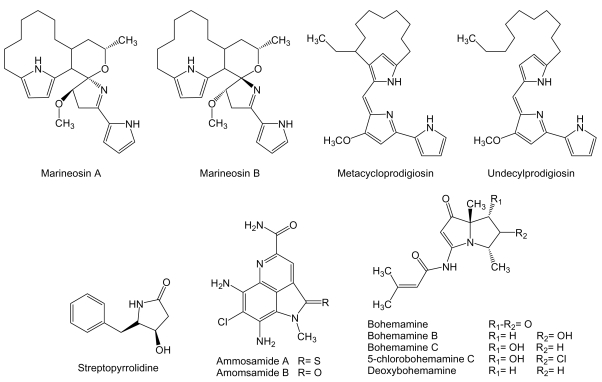
Structures of polypyrrole, tetrahydropyrrole, pyrroloiminoquinone and pyrrolizidine compounds.

Marineosins (Figure 14), related to the prodigiosin class of polypyrrole bacterial pigments, are spiroaminal compounds containing two pyrrole functionalities produced by *Streptomyces* strain CNQ-617 isolated from a marine sediment sample collected off shore of La Jolla, California. Marineosins showed significant inhibition of human colon carcinoma HCT-116 cell line with IC_50_ values of 0.5 *μ*M for marineosin A and 46 *μ*M for marineosin B. These differences might be due to the configuration at the spiroaminal center. In addition, marineosin A showed selectivity against melanoma and leukemia cell lines in the NCI’s 60 cell line panel [156]. Additionally, two known compounds of the prodigiosin family have been isolated from cultures of *Saccharopolyspora* sp. nov. actinomycete isolated from sponge *Mycale plumose* collected along the coast of Qingdao, China [157]. The compounds identified as metacycloprodigiosin and undecylprodigiosin [158,159] exhibited significant cytotoxic activities *in vitro*, as it has been recently described for prodigiosin family of compounds [160], against five cancer cell lines: mouse lymphoma P388, human peripheral blood promyeloblast HL60, lung carcinoma A-549 and SPCA4, and hepatic carcinoma BEL-7402 with IC_50_ values between 0.007 and 7.52 μM for metacycloprodigiosin and 0.013 to 0.11 μM for undecylprodigiosin [157].

Streptopyrrolidine (Figure 14) is a benzyl tetrahydropyrrole derivative produced by *Streptomyces* sp. KORDI-3973 isolated from a deep-sea sediment sample collected at Ayu Trough, in the southern Philippine Sea. Streptopyrrolididine showed anti-angiogenesis activity on human umbilical vein endothelial cells (HUVECs) based capillary tube formation assay. The inhibition of tube formation was exerted without showing cytotoxicity against HUVECs at the concentration of 100 μg/mL [161].

Ammosamides (Figure 14) are pyrroloiminoquinone compounds produced by *Streptomyces* strain CNR-698 isolated from bottom sediments collected at a depth of 1,618 m in the Bahamas Islands. Ammosamide A and B exhibited significant *in vitro* cytotoxicity against human colon adenocarcinoma HCT-116 cells with an IC_50_ of 320 nM each. These compounds were shown highly selective against a diversity of cancer cell lines with values ranging from 20 nM to 1 μM, indicating a specific target mechanism of action that was identified as a member of the myosin family, important cellular proteins that are involved in numerous cell processes, including cell cycle regulation, cytokinesis, and cell migration [162].

Bohemamines (Figure 14) are pyrrolizidine alkaloids produced by *Streptomyces* strain CNQ-583 cultured from a marine sediment sample collected at a depth of 82 m off the island of Guam. Bohemamine, bohemamine B, bohemamine C and 5-chlorobohemamine C were tested for inhibition of the human colon adenocarcinoma HCT-116 cell line but were found to be inactive [163]. However, bohemamine and deoxybohemamine were shown to be inhibitors of cell adhesion based on LFA-1/ICAM-1 capable of inhibiting adhesion of human pro-myelocytic leukemia HL-60 cells to Chinese hamster ovary cells transfected with human ICAM-1, at IC_50_ values of 24.3 and 27.2 μg/mL, respectively [164]. The interaction between LFA-1, also frequently expressed on hematopoietic and solid cancer cell types, and ICAM-1, that in its circulating form promotes angiogenesis and alters cancer cell behavior, is implicated in inflammatory pathologies, autoimmune diseases and in many cancer processes such as cancer metastasis from gastrointestinal carcinoma, melanoma and lymphoma. This interaction is a promising target for the development of new therapeutic agents [165, 166].

**Figure 15:**
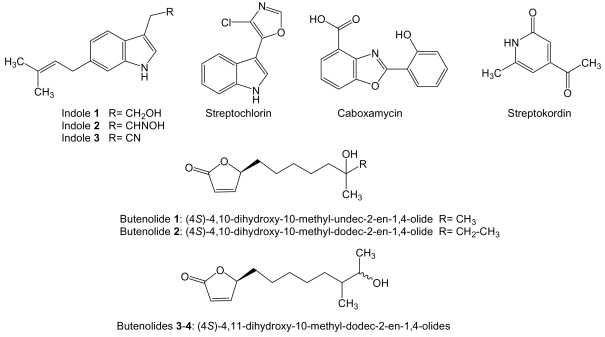
Structure of indole, benzoxazole, methylpiridine and butenolide compounds.

Three new cytotoxic 3,6-disubstituted indoles (Figure 15), with an isoprene unit at carbon 6, were isolated from *Streptomyces* strain BL-49-58-005 isolated from an unidentified marine invertebrate collected in México. These compounds are probably the products of different steps along the same biosynthetic pathway starting from tryptophan or tryptamine as presumed precursors. These indole compounds were assayed against a panel of 14 different tumor cell lines. Indole **1** (6-prenyltryptophol) showed the best activity against human leukemia K-562 cell line with a GI_50_ value of 8.46 μM. The aldoxime indole **2** showed activity with GI_50_ values within μM range against human prostate cancer LN-caP, endothelial cancer HMEC1, leukemia K-562, pancreatic carcinoma PANC1, and colon adenocarcinoma LOVO and LOVO-DOX cell lines. Nitrile indole **3** showed no activity [167]. Streptochlorin (Figure 15) is a 3-substituted indole compound with antiangiogenic and anticancer activities produced by *Streptomyces* strain 04DH110 isolated from shallow water sediment taken at 1 m depth of Ayajin Bay, on the East Sea of Korea. Streptochlorin exhibited significant *in vitro* growth inhibitory activity against human leukemia K-562 cells with an IC_50_ of 1.05 μg/mL [168]. The apoptosis effect induced by streptochlorin was investigated in human leukemic U937 and hepatocarcinoma Hep3B cells showing it was correlated with a decrease in the mitochondrial membrane potential, activation of caspase-3, and down-regulation of antiapoptotic Bcl-2 protein. Those effects were exerted through production of reactive oxygen species in Hep3B cells. In U937 cells up-regulation of pro-apoptotic Bax and FasL, and degradation of poly-(ADP-ribose)polymerase and phospholipase C-γ1 protein was also observed [169,170]. Streptochlorin was found to have, in addition, a potent antiangiogenic activity by inhibition of endothelial cell invasion and tube formation stimulated with vascular endothelial cell growth factor, probably by inhibition of TNF-α-induced NF-κB activation [171].

Caboxamycin (Figure 15) is a benzoxazole compound produced by *Streptomyces* sp. NTK 937 isolated from an Atlantic Ocean deep-sea sediment collected in the Canary Basin. It was tested against different tumor cell lines and showed moderate growth inhibitory activity towards human gastric adenocarcimona AGS, hepatocellular carcinoma Hep G2 and breast carcinoma MCF7 cell lines with GI_50_ of 7.5, 7.4 and 7.3 μg/mL, respectively [172].

Streptokordin (Figure 15) is a methylpyridine compound produced by *Streptomyces* Sp. KORDI-3238 isolated from deep-sea sediments at Ayu Trough. It exhibited significant *in vitro* antitumor activity against human breast cancer MDA-MB-231, colon cancer HCT 15, prostate cancer PC-3, lung cancer NCI-H23, renal cancer ACHN, skin cancer LOX-IMVI and leukemia K-562 cell lines with IC_50_ values raging from 3.2 to 8.6 μg/mL. Streptokordin did not show inhibitory effect at the concentration of 1 mg/mL on the growth of any Gram-positive or -negative bacteria or fungus tested [70].

*S. luteoverticillatum* 11014 [173] isolated from underwater sediment at 20 m depth collected off the coast of Taipingjiao, Qingdao, China, is the producer of four known butenolides: (4*S*)-4,10-dihydroxy-10-methyl-undec-2-en-1,4-olide [174], (4*S*)-4,10-dihydroxy-10-methyl-dodec-2-en-1,4-olide [174,175], and two diastereomeric (4*S*)-4,11-dihydroxy-10-methyl-dodec-2-en-1,4-olides (Figure 15) [175]. The four butenolides showed cytotoxic activity against human leukemia K562 with IC_50_ values of 8.73, 6.29, and 1.05 μmol/mL and murine lymphoma P388 cell lines with IC_50_ values of 0.34, 0.19, and 0.18 μmol/mL, respectively. The mixture of butenolides **3** and **4**, (4*S*)-4,11-dihydroxy-10-methyl-dodec-2-en-1,4-olides, was the most active, but it was unknown if both of the diastereomers were active [173].

**Figure 16:**
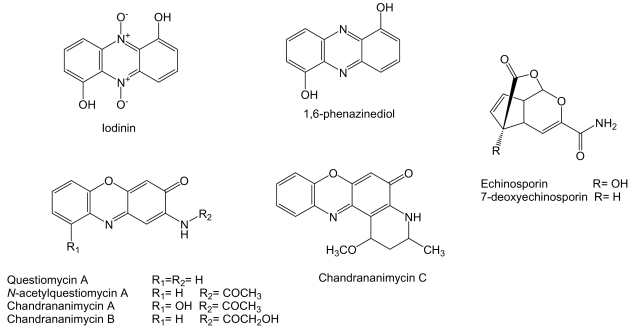
Structures of phenazine, phenoxazin-3-one and tricyclic acetal-lactone compounds.

*Actinomadura* sp. M048 derived from sediments of Jiaozhou Bay in China is the producer of known phenazine compounds iodinin [176] and 1,6-phenazinediol [177], the phenoxazin-3-one compounds questiomycin A and N-acetylquestiomycin A [178], and three new phenoxazin-3-one antibiotics chandrananimycins A, B and C (Figure 16) [179]. Questiomycins and chandrananimycins were found to be active against human colon carcinoma CCL HT29, melanoma MEXF 514L, lung carcinoma LXFA 526L and LXFL 529L, breast carcinoma CNCL SF268, LCL H460 and MACL MCF-7, prostate cancer PRCL PC3M and renal cancer RXF 631L cell lines with IC_70_ values down to 1.4 μg/mL. Iodinin and 1,6-phenazinediol exhibited antitumor activity against the human lung carcinoma LXFA 629L and LXFL 529L, breast cancer MAXF 401NL, melanoma MEXF 462NL, renal cancer RXF 944L and uterus cancer UXF 1138L cell lines with IC_50_ values of 3.6 and 3.2 μg/mL, respectively [179].

Echinosporin and 7-deoxyechinosporin (Figure 16), tricyclic acetal-lactones, are produced by *S. albogriseolus* A2002 isolated from a sea sediment sample collected in Jiaozhou Bay, China [180] and have been previously described as produced by *S. echinosporus* MK-213 [181] and by *Sac. erythraea* Tü 4015 (formerly *S. erythraeus*) where it was shown by feeding experiments that echinosporin is synthesized by the shikimate pathway with chorismate as a biosynthetic intermediate [182]. Echinosporin was shown to inhibit the proliferation of human myelogenous leukemia K562, colon carcinoma HCT-15 and mouse mammary carcinoma tsFT210 cell lines *in vitro* [180], human breast adenocarcinoma MCF7, hepatocellular carcinoma Huh7 and HepG2 cell lines *in vitro* [182] and on rodent tumor models such as leukemia P388, P388/VCR, and fibrosarcoma Meth 1 *in vivo* [183]. 7-deoxyechinosporin showed a weaker effect than echinosporin on K562, HCT-15 and tsFT210 cell lines [180]. Both compounds were shown to arrest the cell cycle of K562, HCT-15 and tsFT210 cells mainly at the G_2_/M phase and to induce apoptosis in these cells [180].

Apart of the antitumor compounds produced by marine actinomycetes depicted above, there are several additional compounds with antitumor activity like the topoisomerase I inhibitors cyclopropane and 14-methylhexadecanoic fatty acids produced by *Streptomyces* sp. strain KM86-913, isolated from a marine sponge collected under the seashore of Keomun Island, Korea [184]. In other cases the compounds identified are yet uncharacterized as is the case of light-activated cytotoxic compounds produced by different microorganisms, including actinomycetes isolated from marine sponges collected from various places in the coast of Peninsular Malaysia [185].

## 8. Conclusions

Actinomycetes and, in particular the genus *Streptomyces*, have been well known during the last seventy years as prolific producers of new bioactive compounds, antitumor drugs included. With the increasing development of oceanographic studies leading to the isolation of new actinomycetes from marine sources, new prolific genera in the production of useful compounds have been found, such as *Salinispora*. However, the Ocean, without any doubt, is keeping a myriad of new actinomycetes providing novel structural diversity to be discovered and used. In addition, the continuous effort for unravel the biosynthesis of the already known compounds and the isolation and characterization of their biosynthesis gene clusters will lead to the development of new antitumor compounds, hopefully with improved therapeutic properties, by using combinatorial biosynthesis approaches.

## Figures and Tables

**Table 1:** Antitumor compounds produced by marine actinomycetes.

Compound	Structural type	Organism	Ref.
1-hydroxy-1-norresistomycin	Polyketide	*Streptomyces chinaensis* AUBN_1_/7	87
		*Streptomyces* sp. B8005	88
1,6-phenazinediol	Phenazine	*Actinomadura* sp. M048	116
1,8-dihydroxy-2-ethyl-3- methylanthraquinone	Polyketide	*Streptomyces* sp. FX-58	86
3,6-disubstituted indoles	Indole	*Streptomyces* sp. BL-49-58-005	167
4a,8a-dimethyl-6-(2-methyl- propenyloxy)- 3,4,4a,4b,5,6,8a,9-octahydro- 1H-phenanthren-2-one	Isoprenoid	*Actinobacterium* sp. MS1/7	143
Actinofuranones	Polyketide	*Streptomyces* sp. CNQ766	65
Altemicidin	Isoprenoid	*Streptomyces sioyaensis* SA-1758	135, 136
Ammosamides	Pyrroloiminoquinone	*Streptomyces* sp. CNR-698	162
Arcyriaflavin A	Indolocarbazole	Actinomycete sp. Z_2_039-2	153
Arenamides	Non-ribosomal peptide	*Salinispora arenicola* CNT-088	117
Arenicolides	Polyketide	*Salinispora arenicola* CNR-005	44
Aureoverticillactam	Polyketide	*Streptomyces aureoverticillatus* NPS001583	53
Bohemamines	Pyrrolizidine	*Streptomyces* sp. CNQ-583	163
Butenolides	Butenolide	*Streptoverticillium luteoverticillatum* 11014	173
Caboxamycin	Benzoxazole	*Streptomyces* sp. NTK 937	172
Chalcomycin	Polyketide	*Streptomyces* sp. M491	49
Chalcomycin B	Polyketide	*Streptomyces* sp. B7064	50
		*Streptomyces* sp. M491	49
Chandrananimycins	Phenoxazin-3-one	*Actinomadura* sp. M048	179
Chartreusin	Polyketide	*Streptomyces* sp. QD518	75
Chinikomycins	Polyketide	*Streptomyces* sp. M045	57
Chlorinated dihydroquinones	Isoprenoid	Actinomycete isolate CNQ-525	141
Cyanosporasides	Polyketide	*Salinispora pacifica* CNS103	66
Daryamides	Polyketide	*Streptomyces* sp. CNQ-085	58
Echinosporins	Acetal-lactone	*Streptomyces albogriseolus* A2002	180
Fridamycin D	Polyketide	*Streptomyces* sp. B6921	80
Griseorhodin A	Polyketide	*Streptomyces* sp. JP95	100, 103
Gutingimycin	Polyketide	*Streptomyces* sp. B8652	84
Himalomycins	Polyketide	*Streptomyces* sp. B6921	80
IB-0028	Polyketide	*Actinomadura* sp. BL-42-PO13-046	98, 99
IB-96212	Polyketide	*Micromonospora* sp. L-25-ES25-008	47, 48
Iodinin	Phenazine	*Actinomadura* sp. M048	179
K252c	Indolocarbazole	Actinomycete strain Z_2_039-2	153
Komodoquinones	Polyketide	*Streptomyces* sp. KS3	73, 74
Lajollamycin	Polyketide/non-ribosomal peptide	*Streptomyces nodosus* NPS007994	132
Lucentamycins	Non-ribosomal peptide	*Nocardiopsis lucentensis* CNR-712	110
Lynamycins	Indolocarbazole	*Marinispora* sp. NPS12745	155
Manumycin A	Polyketide	*Streptomyces* sp. M045	57
Marinomycins	Polyketide	*Marinispora* sp. CNQ-140	54
Marinones	Isoprenoid	Actinomycete isolate CNH-099	137–139
Marineosins	Polypyrrole	*Streptomyces* sp. CNQ-617	156
Marmycins	Polyketide	*Streptomyces* sp. CNH990	94
Mechercharmycins	Non-ribosomal peptide	*Thermoactinomyces* sp. YM3-251	111
Metacycloprodigiosin	Prodigiosin	*Saccharopolyspora* sp. nov.	157
Nonactin	Polyketide	*Streptomyces* sp. KORDI-3238	70
Pacificanones	Polyketide	*Salinispora pacifica* CNS-237	63
Parimycin	Polyketide	*Streptomyces* sp. B8652	82
Piericidins	Polyketide	*Streptomyces* sp. YM14-060	67, 69
Piperazimycins	Non-ribosomal peptide	*Streptomyces* sp. CNQ-593	120
Proximicins	Non-ribosomal peptide	*Verrucosispora* sp. MG-37	107, 109
		*Verrucosispora maris* AB-18-032	108, 109
Questiomycins	Phenoxazin-3-one	*Actinomadura* sp. M048	179
Rabelomycin	Polyketide	*Streptomyces* sp. B6921	80
Resitoflavine	Polyketide	*Streptomyces chinaensis* AUBN_1_/7	87, 93
		*Streptomyces* sp. B8005	88
Resistomycin	Polyketide	*Streptomyces* sp. B8005	88
		*Streptomyces* sp. B4842	88
Saliniketals	Polyketide	*Salinispora arenicola* CNR-005	45
Salinipyrones	Polyketide	*Salinispora pacifica* CNS-237	63
Salinosporamides	Polyketide/non-ribosomal peptide	*Salinispora tropica* CNB-392	64, 121,
		*Salinispora tropica* CNB-440	126
		*Salinispora tropica* CNB-476	122, 131
		*Salinispora tropica* NPS000456	125, 131, 128
Sporolides	Polyketide	*Salinispora tropica* CNB-392	64
SS-228 Y	Polyketide	*Chainia* sp. SS-228	95, 97
Staurosporins	Indolocarbazole	*Streptomyces* sp. KS3	74
		*Micromonospora* sp. L-31-CLCO-002	151
		*Streptomyces* sp. QD518	75
Streptochlorin	Indole	*Streptomyces* sp. 04DH110	168–171
Streptokordin	Methylpyridine	*Streptomyces* sp. KORDI-3238.	70
Streptopyrrolidine	Tetrahydropyrrole	*Streptomyces* sp. KORDI-3973	161
Tetracenomycin D	Polyketide	*Streptomyces* sp. B8005	88
Thiocoraline	Non-ribosomal peptide	*Micromonospora* sp. L-13-ACM2-092	112, 113
T-Muurolol	Isoprenoid	*Streptomyces* sp. M491	49, 140
Trioxacarcins	Polyketide	*Streptomyces* sp. isolate B8652	83
Undecylprodigiosin	Prodigiosin	*Saccharopolyspora* sp. nov.	157
ZHD-0501	Indolocarbazole	*Actinomadura* sp. 007	152
